# Impact of Sensitization of Family Caregivers upon Treatment Compliance among Geriatric Patients Suffering from Elder Abuse and Neglect

**DOI:** 10.3390/healthcare9020226

**Published:** 2021-02-18

**Authors:** Rishabh Garg, Khurshid Mattoo, Lakshya Kumar, Imran Khalid, Fawaz Baig, Mutassim Elnager, Mukram Ali Faridi

**Affiliations:** 1 Department of Prosthodontics, Kalka Dental College, CCSU, Meerut 250005, India; rishabhgarg4u@gmail.com; 2 Department of Prosthetic Dental Sciences, College of Dentistry, Jazan University, Jazan 45142, Saudi Arabia; 3 Department of Prosthodontics, King George Dental College, KGMU, Lucknow 226020, India; lakshya79@yahoo.com; 4 Department of Oral and Maxillofacial Surgery, KKUCOD, Abha 61421, Saudi Arabia; imajid@kku.edu.sa (I.K.); fbik@kku.edu.sa (F.B.); maalnager@kku.edu.sa (M.E.); 5 Department of Maxillofacial Surgery and Diagnostic Sciences, College of Dentistry, Jazan University, Jazan 45142, Saudi Arabia; faridi17@rediffmail.com

**Keywords:** complete denture, elder mistreatment, denture hygiene, treatment compliance, family neglect

## Abstract

Geriatric patients in various outpatient department (OPDs) have been found to agonize from elder abuse and neglect (EAN). Such suffering imposes depressive states within individuals, which in turn affects treatment compliance. The objective of this study was to evaluate the impact of sensitization (psychotherapeutic) of family caregivers (FCGs) upon two denture treatment parameters (maintenance and treatment satisfaction) among EAN patients and compare the differences in outcome with non-abused patients. A survey of completely edentulous subjects (*n* = 860, aged 41–80 years) provided a sampling frame of 332 EAN patients from which 150 patients (including FCGs) fulfilling the study criteria were distributed (simple random, convenient) into two groups (Group A—control, Group B—test). FCG sensitization for subjects in Group B was performed by a clinical psychologist in 2–4 short (30 min) sessions. Demographic characteristics (frequency) were measured using a self-reported questionnaire, denture maintenance was measured using a denture hygiene index (scores), and treatment satisfaction was analyzed on a 10-point visual analog scale. Relevant data were calculated for means and absolute/relative frequencies. Any difference between two groups was estimated using an unpaired *t*-test while the level of relationship was determined by Karl Pearson’s test at a *p*-value of < 0.05. The results showed highest frequency (38.6%) for neglect, with elder neglect (EN) being most common (38.14% alone and 14% in combination). EN was found more if the FCG was a son (52%), in the age group (21–30 years), and with low education and low income (75%). Patients whose FCGs were counselled (Group B) demonstrated low denture plaque scores (mean = 1.38 ± 0.618), while demonstrating comparatively higher scores in six different parameters of treatment satisfaction. Differences between the two groups for both parameters were also found to be statistically significant. Psychotherapeutic counselling in the form of FCG sensitization brings better results of denture maintenance and treatment satisfaction.

## 1. Introduction

Elder abuse (EA) and neglect (EAN) by their respective caregivers (family or non-family members) has unfortunately been ongoing for hundreds of years. Until the advent of initiatives to address various forms of domestic violence (child and spouse abuse) since around 1975, the subject of EA in general remained a personal/private matter, hidden from public view or governmental scrutiny. Initially seen as a highly sensitive social issue and subsequently a problem of ageing, EA received recognition as a public health and criminal justice concern, that subsequently changed the perception of many to rethink how abuse of the elderly should be viewed and/or how it should be analyzed and/or dealt with [1]. Geriatric populations in developing countries have been predicted to increase more than two-fold by 2025 [2], reaching an estimated 850 million people (12% of the developing world’s population), although in particular countries (Colombia, Indonesia, Kenya and Thailand), the surge is projected to be more than four-fold [3]. Contemporary reports by the World Health Organization (WHO) predicts that people aged 60 years and older will increase from 900 million to about two billion by the year 2050. The elderly are the highest users of healthcare systems; therefore, their care in the near future is expected to be challenging. EA basically is a conflict between two adult individuals, where one person is the perpetrator (caregiver, abuser, caretaker) and the other person is a victim (care recipient, abused). The perpetrator can be within one’s own family (Son (most common), spouse, daughter, daughter in law or brother), a relative (within one’s family/in-laws/friends) or can occur in some institute (nurse/staff/resident/professional caregivers/assistant) [4]. EA can occur in various forms such as physical, sexual, emotional/psychological, neglect, financial abuse and/or can also occur in combination [5]. Elder neglect (EN) has been widely reported to be the most common forms, and also difficult to identify irrespective of any range in severity and duration. Similarly to other forms of neglect (e.g., child neglect), EN may be unintentional, while in other cases it may be intentional or deliberate [6]. Dynamics of the relationship between the perpetrator and the victim are poorly understood. On one side, providing care for the elderly can be physically and emotionally demanding [7], although it has also been observed that few people understand the responsibilities and tasks involved in elder care [8]. Healthcare workers, especially physicians, surgeons, orthopedicians, ophthalmologists, nurses and dentists have been designated to play key roles in the identification, reporting and management of EAN in general. Factors such as frequent treatment visits, long appointments leading to trust building, and dynamic communication skills compounded with compassion and empathy have been seen as key indicators for patients to confide/confess their personal sufferings [9]. Gerodontic care involving prosthetic dentures can lead to multiple denture treatment from the same dentist, thus resulting in a very strong and trust-filled doctor–patient relationship [5]. Studies have also reported a high prevalence (30–40%) of EAN patients among the medical [10] and dental [11] outpatient department (OPD), thus inspiring researchers to watch out for the impact of EAN on various medical and dental treatments. Treatment planning for an existing health condition and understanding the influence of EAN simultaneously is a challenging task for healthcare workers, and therefore such an assessment must be as holistic and evidence-based as possible [12].

EA in any of its forms has been shown to impact the victims’ quality of life, and health (medical complications) [13], and even considered as a serious risk of mortality in medical forensics [14]. Psychological stress inflicted by EAN has been strongly associated with the development of depression [15] in elderly subjects. Medical treatments have been shown to exhibit noncompliance in patients with depression [16]. Long-term and complex prosthodontic treatments require patients to be satisfied with the prosthesis to stimulate patient adaptation. Treatment becomes more challenging in EAN patients if a patient is concealing his situation while undergoing a cryptogenic depression within himself. There is also a possibility that although patients may be feeling neglected by their respective family caregivers (FCGs), such neglect may occur unintentionally. Identifying and counselling in such cases, therefore, has to be supplemented to the routine medical or dental treatments to minimize its negative effects. The objective of identifying such psychological stress must be directed to eliminate its cause and/or find a solution, eliminate depression, facilitate self-understanding, and communicate with the self and the counselor [17]. Assertive training for both older persons and their caregivers has been reported to be helpful [18]. This novel, comparative case control study therefore hypothesized the following: (1) geriatric patients in a prosthodontic outpatients department (OPD) do suffer from EAN from their FCGs; (2) psychotherapeutic intervention (FCG’s sensitization) improves the patient’s ability to maintain prosthesis and subsequently enhance treatment satisfaction; and (3) differences in denture maintenance and treatment satisfaction (dependent variable) will exist after such an intervention (independent variable).

## 2. Materials and Methods

### 2.1. Ethics

This study was conducted within the geriatric care unit of the department of oral and maxillofacial prosthodontics under the observation of the college and the university ethics committee of a medical university in northern India. All human and animal studies were conducted following strict adherence to the ethical principles and standards of the Helsinki Declaration. All participants were routine OPD patients (eligible elderly subjects), along with their respective FCGs (blood-related). All subjects were briefed about the beneficence of the study, and after assuring their confidentiality, a written informed consent was obtained.

### 2.2. Study Design

This comparative, case control study was conducted between September 2017 and January 2020, on a sample of the North Indian population. The study was accomplished in three stages. The first stage was a large-scale survey among completely edentulous (CE) patients (aged 41 to 80 years) that provided the sample and subsample for the actual study. The second stage involved fabrication of complete dentures (CD) treatment, and the final stage was the follow-up of all patients one month after denture insertion. The study implemented both qualitative and quantitative approaches at different stages to analyze the obtained data.

### 2.3. Operational Definition

In the context of the present study, EA was defined as an act or omission that may result in harm or threatened harm to the health and/or welfare of an elderly individual. [4] EN was referred to as the failure of the caregiver to meet the older adults’ basic needs (food, water, shelter, clothing, hygiene and essential medical care) [19]. Although certain researchers draw certain distinctions among abuse and neglect, this article intends to use the term interchangeably and inclusively (as EAN), because most of the explanations have been addressed to EA in particular. The family caregiver was defined operationally as an individual who, as a result of family relationship (i.e., blood-related), was accountable for caring the concerned elderly adult [7]. Terms such as victim, abused, and care recipient are synonymous with the person who has been abused, and terms such as perpetrator, abuser, caretaker are synonymous to the FCG involved in abusing the elderly.

### 2.4. Sample Preparation, Selection and Grouping

In the first stage of this study, 860 CE patients in the Prosthodontics OPD were screened for the presence/absence of dementia and/or any cognitive impairment using an elderly cognitive assessment questionnaire (ECAQ), [20] and existence of elder abuse using the Elder Abuse Suspicion Index (EASI) [21]. The screening was conducted by a four-member team that included a psychiatrist, clinical psychologist, physician, and a prosthodontist. A total of 332 patients at this stage were found to be suffering from elder abuse (suspicious), who were then further screened for fulfilling the criteria of the main study. Inclusion criteria for patients were those seeking CD treatment for the first time, living with their family (children), not suffering from any systemic diseases (physical or mental), no hearing or vision loss, no history of drug/alcohol use, willing and cooperative patients (i.e., would share personal sensitive information in relation to treatment outcome, ready to undergoing long duration treatment, understand their role in the denture maintenance). Essential criteria for being considered as an FCG included the caregiver being willing to accompany the concerned patient, not having any history of aggressive/violent behavior, having no history of drug/alcohol dependance, not having any caring demands from their immediate family (spouse/children) themselves, not being medically/physically/mentally compromised, no history of being neglected by their parents during their childhood, and not being dependent on the elderly patient. Patients who were excluded included old denture wearers, showing signs of self-neglect (dirty clothing, unhygienic hair and nails, foul body odor), childless patients, those who had lost a child or children after marriage (natural or unnatural cause), and patients with poor edentulous foundation/poor neuromuscular control and neuromuscular coordination. The CD prosthetic treatment was standardized in terms of total number of appointments (14 to 16 appointments), clinical and laboratory procedures, and prosthesis designing. All patients were allotted to postgraduate students who were supervised by a team of two prosthodontists and a clinical psychologist (experience ≥8 years). All clinicians were reviewed regarding various trust-building measures and cautious approach that needed to be exhibited in order to verify the existence of elder abuse among their respective allotted patients. A detailed questionnaire that investigated the existence of EAN by asking indirect questions during the interview was incorporated within the case history recording for CD fabrication. During subsequent treatment appointments, 312 patients out of a total of 332 (93.9%) patients, confided (verified) to their respective clinicians that they were suffering from EAN by their respective FCGs. During this verification stage of their respective EA status, the patients were subsequently and simultaneously designated into two groups, Group A—control group (FCG not counselled) and Group B—test group (FCG counselled). The optimum number of subjects that fulfilled the requirement of efficiency, representativeness, reliability, and flexibility for test group was decided to be 75 patients (subsample) in each group (± 5% accuracy, an alpha of 0.05 (95% confidence interval)). We were working with a sample that has been derived from a particular population; therefore, the goal was to describe and draw inferences regarding the studied population. In such a case, the confidence interval (CI), a descriptive statistic measure, can be used to draw inferences regarding the studied population [22]. An equal number of males and females (64 males, 11 females) were randomly distributed in both groups. Sample design for the subjects of both groups was convenient (consecutive) sampling (convenience being the existence of EAN) with replacements. All individual patients were treated by the same doctors and assistants throughout the treatment. All CD prostheses upon completion were evaluated by a separate team of experienced prosthodontist for prosthetic quality. Patients whose prostheses were unsatisfactory were not included in the study and were recommended for a new prosthesis. All patients in both groups received verbal and written post-insertion instructions and a denture maintenance kit (denture brush, denture cleanser tablets, denture box) for long term maintenance. All respective patients in both groups were recalled after one month for objective evaluation of denture hygiene (denture hygiene index) and treatment satisfaction (scaled questionnaire).

Counselling of FCGs: caregiver sensitization (psychotherapeutic) for patients in Group B was conducted during the last stages of CD fabrication and was performed in a private setting by a clinical psychologist. The counselling sessions of FCGs were kept short, approximately 20–30 min. The number of counselling sessions ranged between 2 to 4 (depending upon the clinical judgement of the clinical psychologist for counselling to be effective). Cases of unintentional EAN required minimum counselling, while intentional neglect cases required more counselling sessions. All FCGs were counselled in the absence of their respective care recipients, except the last session, where both were counselled for steps and roles of both parties in enhancing the patient’s adaptation to the CD prosthesis. Basic principles of counselling were strictly followed so that the relationship between the patient and their caregiver did not deteriorate. During the counselling, the significance of the caregiving stress of FCGs was discussed for each individual, and the strategy to overcome such stress was communicated to each FCG during counselling sessions. The caregivers were encouraged to express their concerns related to caring for the elderly. FCGs were stimulated to reveal whether the caregiving was forced upon them or they volunteered to do so, despite other members in the family being there to take care of the elderly. The first counselling session aimed to gather maximum information about the FCG and their relationship with the elderly person, while the concluding sessions were focused primarily on educating the FCG as to how to make providing care a pleasing experience. The FCG was also educated about how to read the elderly person’s mind and how to defer elders’ expectations, rather than outrightly reject them out of anger. Motivation was also directed to make the caretaker believe that patients’ existing overall conditions needed utmost care and cooperation of all their family members, and that they need to be the fulcrum (putting the entire responsibility on the FCG) in changing the patient’s lifestyle and attitude towards prosthetic care.

### 2.5. Measures, Data Evaluation, Collection and Analysis

Demographic characteristics of patients and their respective FCG were acquired using a self-administered structured questionnaire by each patient. Different versions (English, Hindi, Urdu, Sanskrit) of the questionnaire were prepared to enhance clear communication. Information regarding the type, severity, and duration of EAN were filled by the concerned postgraduate student who was treating the case. Each treating doctor was asked to maintain a diary, in which all discussions between them and their patient were to be included. A special note was kept on the day when the patient would accept the existing of EAN by their FCG. Counselling of the caregivers was recorded as a report for each patient. The denture plaque index was used as an indicator to evaluate the patients’ motivation and compliance. The disclosed denture plaque on the denture was scored as described in the literature (Table 1) [23]. Denture hygiene scores were evaluated one month post-insertion of the complete dentures for all subjects in both groups. For evaluation of subjects’ treatment satisfaction, a questionnaire was answered and quantified on a 10-point visual analog scale (VAS). The scale presented a list of questions in a random question sequence with a reversed polarity of questions, as previously reported [24]. The patients evaluated their dentures by using the VAS. All filled questionnaires were collected, reviewed, coded, and studied, (Supplementary data) and then relevant data analysis was completed using the Statistical Package of Social Sciences (SPSS 25.0) (IBM, Armonk, NY, USA) software. Mean values ± standard deviations (median with interquartile range) were used for continuous variables, while relative and absolute frequencies for qualitative variables were calculated. The difference between the two groups for denture hygiene and for the eight parameters of denture satisfaction was done using an unpaired *t*-test, while Karl Pearson’s test was used to determine the level of relationship between linear related variables. All differences were considered to be significant at a *p*-value of < 0.05.

## 3. Results

**Figure 1:**
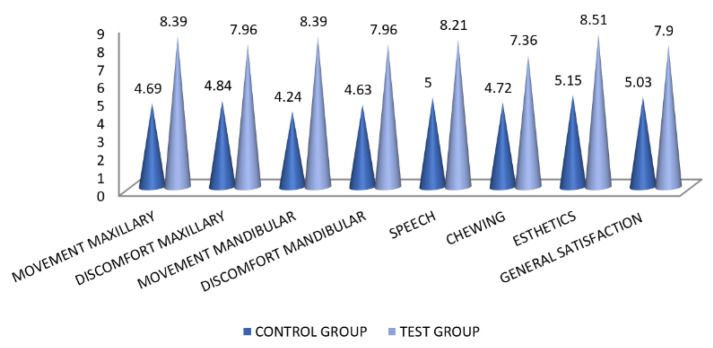
Distribution of mean scores for various parameters of denture satisfaction between the two groups.

The present study involved an initial survey, from which a study sample was selected for the main study. Comparative demographic data for the sample frame and the sample are presented in Table 2. A higher frequency of abused elderly was found for ages 50 to 60 years (37.5%) and 60 to 70 years (30%). Elder neglect (38.14%) was the predominant type of EA within the subjects and was also found to be a common type of abuse in the combined abuse type (14.10%). A higher frequency of elder abuse was found in subjects who had low education (illiterate, primary school). Caregiver characteristics in the survey found sons to be primary abusers, followed by daughters-in-law. Out of a total of 312 FCGs (61% males, 39% females), 18 scheduled FCGs did not accompany the elderly due to their own work. All the patients were living with their respective children and none were living with relatives or in old age homes. A higher frequency of elder abuse was found where the FCG’s age was between 21 and 30 years (51.9%), the FCG was illiterate (68.9%), and those whose income was low (75.32%). The comparative demographic characteristics of respective patients and their FCGs that were allotted in two different groups are shown in Table 2. Individual scores (frequency distribution) and mean scores obtained on denture the plaque index among subjects of both groups is shown in Table 3. Subjects belonging to Group B (mean = 1.38 ± 0.618) had lower scores of denture plaque than subjects of Group A (mean = 2.92 ± 0.892), indicating better hygiene maintenance by patients of Group B. The differences between the two groups were statistically significant at a *p*-value ˂ 0.05. There was, however, no significant relationship between two linearly related variables (changes in the values of one variable causing a proportionate linear change in the values of the other variable). Treatment satisfaction was measured for eight different parameters for the CD prosthesis (movement of individual dentures, comfort, speech, ease of chewing, esthetics, and general satisfaction) on a visual analog scale (from 1 to 10, with 1 being the lowest and 10 being highest). For all parameters of denture satisfaction, the mean values were found to be higher among subjects in Group B than Group A (Table 4) (Figure 1). Statistically significant differences between the two groups were found in all parameters of denture satisfaction at a *p*-value of less than 0.05. The degree of correlation between linearly related variables was found to be statistically significant for three treatment satisfaction parameters, namely the movement of mandibular denture, speech and general (overall) satisfaction. 

## 4. Discussion

This study involved two clinical stages, an analytical survey to find the existence of EA among patients in the OPD, and an interventional, comparative case control stage, in which the intervention was in the form of counselling (psychotherapeutic) of FCGs of each individual subject selected in the study. The study reports that in a prosthodontic outpatient department (where the majority of patients are elderly), 38.6% of elderly patients (aged 41 to 80 years) were found to be suffering from EA, with neglect being most common (38%). EN was found to be more associated among those who were illiterate or less educated, where FCG’s age was between 21 to 30 years (51.9%) and FCG’s income was low. FCG’s kinship with the neglected elderly were mostly either a son (52%) or daughter-in-law (31%). The main findings of the study include improved denture hygiene scores among the patients in Group B, whose FCG’s were counselled (mean = 1.38) as compared to patients in Group A (mean = 2.92). Eight parameters for denture satisfaction were investigated, and the results showed significant differences in treatment satisfaction between the two groups. The three parameters of denture satisfaction (movement of mandibular denture, speech, and general satisfaction) showed a linear relationship between the two studied variables. The overall results suggest that patients who are suffering from neglect from their caretakers show decreased treatment compliance (satisfaction) to prosthodontic treatment.

Elder abuse is a social phenomenon which may have existed for centuries across all cultures, ethnic groups and among all religions, although evidence in medical and social practice was not found until the late 1990s. Although its prevalence rate has been estimated to be 16% on average in a community setting [25], its occurrence in dental outpatient departments (40%) and emergency departments (55%) has been variable [10,11]. The higher frequency seen in the OPDs could be because more elderly patients regularly seek medical help compared to young people. This study was conducted in North India, where religious, cultural, and traditional practices are extremely respectful towards their elderly [11]. Irrespective of the elders’ attitude towards their children, the society generally expects that young people have to take care of their parents properly. Social relationships (e.g., marriages) are largely based on the involvement and assurance of parents rather than the spouse.

As per the latest population census of the region (2011), a total of 104 million old age population groups were recognized, with almost equal gender distribution [26]. The most common abuse perpetrator (son) is in agreement with various international [27] and national studies [28]. However, a study among medical college hospital patients in southern India reported a prevalence of only 16% (*n* = 200) [29] as compared to 38% (*n* = 816) reported in our study. The differences are understandable because there are many younger patients who report to a medical hospital, while most of the patients reporting to a prosthodontist are elderly. Another reason could be the differences in the sample size between the two studies. A third explanation is that the education level and the expenditure on education by the population between the two regions (north/south) varies considerably [4]. Neglect of the elderly is largely associated with Asian cultural practices of living with their son (primarily the eldest son) while being poor and dependent within the family.

### 4.1. Ever-Changing Role of Healthcare Workers (HCW) in Detecting Elder Abuse

Despite EAN being a social problem (law and order in the case of physical/domestic abuse) for many years, it is the HCWs who are primarily responsible for identifying and reporting, because it is mostly hidden or intended to be kept hidden by both victim and the perpetrator. Medical professionals, especially family physicians/family dentists and nurses, have been thought of to be in a better position to detect EAN, because they tend to develop a trust/relationship over a period of time because of frequent patient visits [14]. It has been reiterated in the scientific literature that hospital screening is significant because it could be the only place of contact with the victim and the perpetrator outside of their residence [30]. While HCWs may consider it as a professional responsibility to identify and intervene in cases of EA, it is also true that both doctors and nurses have been found to have deficient knowledge of indicators of EA [31,32]. Most of the HCWs lack training to identify EA. In order to elicit EA, one needs to develop skills in one’s attitude, scrutinizing ability, the following of ethics related to EA, and history taking. In the context of the present study, the need of discussing this parameter is to highlight the status of HCWs in terms of recognizing EA. HCWs who were involved in this study were first trained by clinical psychologists before they could be exposed to such a sensitive issue in their patients. The line of training under various parameters has been summarized in Table 5.

The results of our study show that 38.6% (*n* = 860) of patients visiting a prosthodontist were suffering from EA in one or many forms, thus reiterating the significance of creating awareness among HCWs. All postgraduate students and their supervisors needed to be trained to elicit the existence of EA among their patients. HCWs have been reported to just witness infrequent discussions rather than the actual reporting of EA. This is due to existence of multiple barriers (lack of protocols/training, communication challenges, time limitations, lack of follow up) [32]. At the same time, there are studies that implicate EA victims with increased risk of mortality and developing adverse health problems (depression, disability, hospitalization) [33,34]. Identification of dynamic risk factors have also paved the way for HCWs to intervene rather than merely report [35].

### 4.2. Need of Recognizing the Clinical Impact of EAN on Routine Medical/Dental Treatment Plans

EAN victims, especially female patients, have been reported to consult medical practitioners for different clinical conditions that are either direct or indirect consequences of EAN [36]. It is obvious that EAN victims are not expected to consult medical practitioners specifically for preventing the abuse. Medical conditions that develop as a consequence of EAN, such as depression, anxiety, high blood pressure and heart problems, have also been shown to have a direct impact on one’s general health [37]. Apart from the effects (direct/indirect) on one’s health, studies show that the chances are three-fold higher for a depressed patient to be non-complaint to medical treatment than a non-depressed patient [16], explaining why medical treatments fail in such cases. The problem becomes more complex if the EAN victim has an underlying systemic problem (debilitating) not related to EAN, because there is greater likelihood of patients to develop depression with underlying health problems [38]. In prosthodontics, the denture satisfaction has also been observed to be inversely proportional to the patient suffering from depression. The probability of denture satisfaction has been observed to decrease by 24% with each unit increase on a 15-point depression scale [39]. The relationship between depression, EAN, and treatment satisfaction justifies the need of this study while indirectly providing the explanation of the results as well. Our results further reiterate the importance of identifying the EA before starting treatment. The patients in the test group had better treatment satisfaction and improved denture hygiene, because there was improvement in the relationship between the FCG and the abused elderly person.

### 4.3. FCG’s Parameters and Caregiving

The etiology of EN is complex and variable depending upon its type. There are multiple theories that explain its occurrence [1,4,11]. While early theories postulated that the elders’ dependance on the caregiver results in caregiver stress, contemporary studies have stated that it is the caregivers’ dependance on the victim that is a dominant risk factor for EAN [40]. Such claims have been further substantiated by studies who identified unemployment, financial dependence of caregivers, and poverty as risk factors for EAN [41]. A meta-analysis by Johansson, however, ruled out financial dependency as a risk for caregiver elder abuse [42]. Irrespective of social parameters, there is little doubt that caregiving at any level is demanding and therefore stressful. Caregiving for a physically dependent elderly person may include various types of care (personal, household, emotional and healthcare). Caregiving for the elderly has been found to affect the health and social life of caregivers [43,44]. Results of FCGs from this study show that the EN was mostly committed by either a son or daughter-in-law, which is in agreement with earlier studies of the region [5,26,28]. The obtained data also show that the majority of the FCGs associated with EAN were less than 30 years old (65%), had low income, and with a poor education background. Young FCGs may not be able to overcome the stress related to caregiving. Young caregivers may also be unemployed, which is directly associated with having low income. Poor educational attainment among unemployed caregivers has been reported to result in high caregiver stress as compared to those who were employed or more educated [45]. Studies have also reported that caregivers with high income were more likely to experience less stress as compared to those with low income [46], while there is only one study that reported more stress among high income caregivers [7]. Stress coping has been also found to be different across different races and ethnicity [47].

### 4.4. EAN and the Type of Intervention

Before any type of intervention is undertaken in the case of EA or EN, it is imperative for everyone to differentiate EN from self-neglect and unintentional (unaware caregiver) neglect because interventions differ accordingly. Manifestations of EN and self-neglect may be similar; therefore, in such cases a person’s unwillingness to improve their basic needs for physical, emotional, and social well-being, differentiates the two types of neglect [48]. Intentional neglect also needs to be differentiated from unintentional neglect because caregivers may be very caring for the elderly but may not know about the health status of the elderly. It is also important to differentiate between an FCG deliberately enforcing EAN or not, because intervention of any sort can further deteriorate the relationship between the caregiver and the elderly. Intentional EAN of caregivers should be established either directly or indirectly (elder acceptance) before any intervention. It has been emphasized frequently by leading researchers to develop, evaluate, and scale up potentially effective prevention intervention strategies and approaches to curb EA [49]. Until a system is developed where interventions to stop EAN will work, we might as well use a masked intervention as employed in this study. For a medical/dental practitioner, cautious approaches to elicit and/or intervene are desired, the principles of which have been summarized in Table 5. The intervention which was undertaken in this study was a psychotherapeutic type [50], in which individual counselling was given to FCGs by trained professionals. The counselling was designed to teach the FCGs the coping skills that were required to help the patient adapt to new complete dentures as quickly as possible to ensure treatment satisfaction. The onus was primarily to make the FCG believe that without their help, it would be difficult to achieve treatment objectives. Rather than blaming them for neglecting the elderly, they were given the responsibility to encourage their remaining family members to take care of the elderly person. This was seen as a new way to rejuvenate the relationship between the elderly patients and their FCGs. According to the results of this study, completely edentulous patients whose FCGs were counselled, were observed to have low denture plaque index scores (mean = 1.38) after one month of evaluation, as compared to those whose FCGs were not counselled (mean = 2.92). On the contrary, studies of educational intervention given to nursing caregivers (staff) indicated no improvement of oral or denture hygiene [51]. The differences could be primarily due to the type of caregiver (family caregiver instead of a nurse) and sensitization (individual against group) in this study. Another possible reason could be that we included only neglected subjects, because we believed that other forms of elder abuse would not fit in one type of intervention method and might further affect the elder–caregiver relationships. Studies in the past, however, have associated poor oral/denture hygiene and root caries with EN [52]. Other orofacial manifestations that should suggest EAN are a lack of needed dentures, old dentures not being replaced, improper old dentures, dry mouth, and mucosal lacerations. Patients who suffer from EAN most commonly suffer from depression, which clinically manifests as dissatisfaction/boredom with one’s life, being helpless, or feeling worthless [15]. Underlying depression prohibits an EN patient from complying with treatment recommendations; in other words, such patients have been found to demonstrate non-adherence to their treatment (indifferent behavior) [53]. One of the examples of indifferent behavior is not to clean dentures as recommended, or not following the instructions that have been recommended for using the prosthesis.

## 5. Strength and Limitations of the Study

The study highlighted the significance of identifying EAN, among patients (both medical and dental) and its association with patient compliance in terms of complete denture prosthesis (denture maintenance and denture satisfaction). The study also guides a clinician as to how to identify cases of EAN and different interventional strategies that could be employed. The main strength of the study was its method, in which the patients had verified the existence of EAN from their FCGs. However, this method of verifying the existence of EAN made this study time-consuming. Limitations include the study only applying to family caregivers of elderly people with a known history of EAN, and therefore are only being applicable to FCGs with similar characteristics. The results are therefore only applicable in cases of neglect, and cannot be applied to other types of abuse. Other limitations include the cross-sectional design (findings only imply associations and not causal relations), and not being applicable to institutionalized elderly and/or professional caregivers.

## 6. Conclusions

Patients visiting a prosthodontist seeking complete denture prosthesis do suffer from EAN. A prosthodontist must include screening for EAN, because such patients tend to suffer from depression. Postgraduates in prosthodontic care should be taught how to elicit and intervene in such cases. Psychotherapeutic intervention of FCGs should be performed after differentiating types of neglect. Sensitization of caregivers should be considered as routine in all cases, the goal of which is to improve patient compliance by empowering their FCG with the responsibility of the elderly patient. Long-term studies extending to evaluate after at least one or two years need to be conducted.

## Figures and Tables

**Table 1:** Denture hygiene scoring criteria.

Score	Grade	The Total Amount of Plaque Disclosed on the Denture Surface
1	Good	No plaque
2	Average	Moderate plaque (25% to 50% of the fitting surface and tooth surface was covered)
3	Poor	Heavy plaque (51% to 75% of the fitting surface and tooth surface was covered)
4	Very poor	Very heavy plaque (76% to 100% of the fitting surface and tooth surface was covered)

**Table 2:** Sociodemographic characteristics of elderly subjects (main survey) and sample subjects (control/test groups).

Characteristic	Parameters	Main Survey *n* (%)	Sample Subjects *n* (%)	
			Group A (Control)	Group B (Test)
Prevalence	Total number of subjects	*n* = 860	*n* = 75	*n* = 75
	Suspected elder abuse (EASI)	332 (38.6)	N.A.	N.A.
	Confirmed (self-revelation)	312 (36.27)	N.A.	N.A.
Gender (abused)	Male	198 (63.4)	64 (85.33)	64 (85.33)
	Female	114 (36.5)	11 (14.67)	11 (14.67)
Age distribution of abused (in years)	41–50	42 (13.46)	1 (1.33)	3 (4)
	51–60	117 (37.5)	26 (34.66)	32 (42.67)
	61–70	94 (30.12)	26 (34.66)	20 (26.67)
	71–80	59 (18.9)	22 (29.33)	20 (26.67)
Abuse type distribution	Neglect	119 (38.14)	59 (78.66)	51 (68)
	Psychological	53 (16.98)	N.A.	N.A.
	Financial	31 (9.93)	N.A.	N.A.
	Physical	64 (20.51)	N.A.	N.A.
	Sexual	1 (0.32)	N.A.	N.A.
	Combination (neglect and one or more)	44 (14.10)	16 (21.33)	24 (32)
Level of education of abused subjects	Illiterate/primary school	267 (85.57)	62 (82.67)	68 (90.67)
	Literate/secondary school/graduate/postgraduate	45 (14.42)	13 (17.33)	7 (9.33)
Abuser (family caregiver) types (*n* = 312)	Son	165 (52.88)	41 (54.66)	42 (56)
	Daughter-in-law	97 (31.08)	26 (34.66)	23 (30.6)
	Spouse	24 (7.69)	8 (10.67)	6 (8)
	Brother/Sister	12 (3.84)	0 (0)	2 (2.66)
	Others	2 (0.65)	0 (0)	2 (2.66)
Age distribution of family caregiver	≤20	45 (14.42)	5 (6.67)	6 (8)
	21–30	162 (51.9)	31 (41.33)	44 (58.6)
	31–40	90 (28.84)	34 (45.33)	20 (26.67)
	41–50	10 (3.2)	4 (5.3)	2 (2.66)
	≥51	5 (1.6)	1 (1.33)	3 (4)
Income of family caregiver *	Low	235 (75.32)	56 (74.6)	50 (66.67)
	Average	65 (20.83)	12 (16)	20 (26.67)
	High	12 (3.84)	7 (9.34)	5 (6.67)
Level of education of family caregiver	Illiterate/primary school	215 (68.9)	57 (76)	59 (78.67)
	Literate/secondary school/graduate/postgraduate	97 (31.08)	18 (24)	16 (21.34)

*n*, number of subjects; %, value expressed in terms of percentage within parenthesis; EASI, Elder Abuse Suspicion Index; N.A., not applicable; *, income described as per the World Bank country classification (Atlas method).

**Table 3:** Denture plaque index scores (DPI) with means compared among subjects in both groups.

Scores	Group A (Control)				Group B (Test)				Probable Value of *t* (Unpaired)	Karl Pearson Correlation Coefficient (*r*)
	*n* (75)	%	Mean ± SD	SEM	*n* (75)	%	Mean ± SD	SEM	0.0000 *	−0.0017 (NS)
Good	4	5.3	2.92 ± 0.892	0.1564	52	69.33	1.38 ± 0.618	0.1084		
Average	23	30.6			18	24				
Poor	27	36			5	6.67				
Very Poor	21	28			0	0				

In the unpaired *t*-test, the level of the degree of significance was determined on the value of *p* < 0.05; degree of linear relationship between two variables was determined by the Karl Pearson’s correlation coefficient, expressed as *r*; * significant; NS, not significant.

**Table 4:** Comparative differences between the two groups studied for various parameters of denture satisfaction.

	Group A (Control)		Group B (Test)		Probable Value of *t* (Unpaired)	KP Correlation Coefficient (*r*)
Parameters	Mean ± SD	SEM	Mean ± SD	SEM		
Movement Maxillary Denture Comfort Maxillary Denture Movement Mandibular Denture Comfort Mandibular Denture Speech Ease of chewing Esthetics General Satisfaction	4.69 ± 1.286	0.2256	8.39 ± 0.658	0.2256	0.0000 *	0.0715
	4.84 ± 1.372	0.2407	7.96 ± 0.918	0.2407	0.0000 *	0.1699
	4.24 ± 1.031	0.1808	8.39 ± 0.658	0.1808	0.0000 *	0.0390 *
	4.64 ± 1.245	0.2184	7.96 ± 0.918	0.2184	0.0000 *	0.1465
	5 ± 1.118	0.1961	8.21 ± 0.780	0.1961	0.0000 *	0.0002 *
	4.72 ± 1.125	0.1973	7.36 ± 0.895	0.1973	0.0000 *	0.1946
	5.15 ± 1.121	0.1966	8.51 ± 0.667	0.1966	0.0000 *	−0.2329
	5.03 ± 1.103	0.1935	7.90 ± 0.630	0.1935	0.0000 *	0.0408 *

In the unpaired *t*-test, the level of the degree of significance was determined on the value of *p* < 0.05; degree of linear relationship between two variables was determined by the Karl Pearson correlation coefficient, expressed as *r*; * significant differences.

**Table 5:** Principles for eliciting/intervening in elder abuse and neglect.

Parameter	Clinical Guidelines
Attitude	Patience and tolerance during history taking Clear and slow speech (words) that have low tone Do not infantilize the patient Do not subscribe patient to any ageing myth or attitude (forgetfulness, dependency, unproductivity, unattractiveness) Respect ethnic, cultural, and religious differences between the patient and yourself Do not disbelieve in what patients say (initially, patients will deny any harm done by someone with them) Interview the patients in the absence of a caregiver Use questions that are neutral and non judgmental in nature Do not allow caregiver to answer questions for the patient
Scrutiny	Cuts, bruises, lacerations, dehydration, nutritional deficiency, weight loss, burns Pay close attention to patient–caregiver interactions (verbal and non-verbal—discomfort, silent patient, monosyllabic responses, anxiety, palpitation) Observe the patients’ behavioral responses and body language (fear, disorientation while responding, anger, infantile behavior, agitation, sucking) Look for confusion, withdrawal, frequent denial, implausible tales, and failing to talk openly
Ethics	Respect options and choices patient make regarding their situation or about the caregiver Provide a source of emergency assistance and or a safety plan if the need arises Any intervention by a psychologist should be after consulting the patient and the patient’s doctor before deciding any course of action All decisions should be made on weighing the beneficence and maleficence
History taking	Look for clues of transgenerational violence (history of domestic violence as perpetrator or victim) Past relationships (death of spouse, partner, child) Family dynamics (number of household members, sources of income, resources)Education levels, employment, and financial status, Substance abuse among patient and/or family member Sexual history
Intervention	Psychoeducational—improves caregiver’s knowledge about themselves, care recipients, and the environment. Focus on teaching caregiver to develop skills to deal with stress Psychotherapeutic—involves individual counselling to caregivers by trained professionals and teaches coping skills and problem solving Supportive—to build support systems or networks among caregivers and create an environment for them to discuss and share Service based—facilitate caregivers’ use of formal services, improving competence of care recipients and delaying institutionalization of care recipients

## Data Availability

Mentioned in Supplementary Materials.

## Supplementary Materials

The following are available online at https://www.mdpi.com/2227-9032/9/2/226/s1.
